# Low-protein diets supplemented with glycine improves pig growth performance and meat quality: An untargeted metabolomic analysis

**DOI:** 10.3389/fvets.2023.1170573

**Published:** 2023-04-18

**Authors:** Shengwang Jiang, Wei Quan, Jie Luo, Aihua Lou, Xihong Zhou, Fengna Li, Qingwu W. Shen

**Affiliations:** ^1^College of Food Science and Technology, Hunan Agricultural University, Changsha, Hunan, China; ^2^College of Animal Science, Xichang University, Xichang, Sichuan, China; ^3^Institute of Subtropical Agriculture, Chinese Academy of Sciences, Changsha, Hunan, China

**Keywords:** low protein diet, glycine, metabolomics, growing-finishing pigs, meat quality

## Abstract

For the purpose to improve meat quality, pigs were fed a normal diet (ND), a low protein diet (LPD) and a LPD supplemented with glycine (LPDG). Chemical and metabolomic analyses showed that LPD increased IMF deposition and the activities of GPa and PK, but decreased glycogen content, the activities of CS and CcO, and the abundance of acetyl-CoA, tyrosine and its metabolites in muscle. LPDG promoted muscle fiber transition from type II to type I, increased the synthesis of multiple nonessential amino acids, and pantothenic acid in muscle, which should contributed to the improved meat quality and growth rate. This study provides some new insight into the mechanism of diet induced alteration of animal growth performance and meat quality. In addition, the study shows that dietary supplementation of glycine to LPD could be used to improved meat quality without impairment of animal growth.

## 1. Introduction

China is the largest pork producer and consumer in the world, of which the production and consumption reached 54.04 and 55.95 million tons in 2018 according to the National Bureau statistics,^1^ respectively. However, with the expanding breeding of the imported commercial crossbreeds, the meat quality is being increasingly criticized by consumers. Obviously, this is mainly due to the reduced intramuscular fat (IMF, commonly below 2.5%) resulted from genetic selection for lean growth (1, 2), as IMF greatly affects meat quality and some early studies showed that the sensory traits of pork, like juiciness, tenderness and overall acceptability, are negatively affected when IMF content is lower than 2 ~ 2.5% (3). Conversely, autochthonous, rustic pig breeds commonly present higher subcutaneous and IMF content, which are scored higher by sensory panels (4, 5). Thus, strategies have to be developed to ameliorate meat quality.

Reduced protein diets (RPD), alone or combined with some components, such as function amino acids, seems to be a promising approach to satisfy consumer requirements for meat quality with no increase in or even lower production costs to the livestock sector (1). A number of studies have shown that dietary protein reduction to 15–20% of NRC increases IMF content in the growing-finishing pigs (1). Dietary protein restriction (11.9, 13.3, 14.8, 16.2 and 17.6%) increases marbling and IMF content of *longissimus dorsi* muscle of finishing pigs, with the lower dietary protein concentrations showing the higher marbling and IMF content (6). In addition, reducing crude protein concentrations from 19.0 to 10.0% in a diet fed to pigs of 30–110 kg body weight increases IMF content of *longissimus dorsi* muscle from 3.8 to 9.4% (7). At the same time, Kerr et al. (8) reported that dietary protein restriction from weaning until market weight increased IMF content in the *longissimus dorsi* muscle of pigs to a two-fold level than that of control pigs. The effects of RPD combined with amino acids on animal performance and meat quality have also been studied. Previous literature reports that dietary supplement of arginine promotes muscle growth and IMF deposition, but decreases whole body fat content in pigs (9, 10), showing improved fat partitioning. Compared with the normal protein diet, the RPD and RPD supplemented with arginine increased IMF of pigs from 1.34 to 1.85 and 2.30 g/100 g muscle, respectively, and consequently improved meat tenderness and overall acceptance (11). Although RPD with leucine supplementation had no effect on IMF content (12), it increased meat juiciness (11). All these studies demonstrate that dietary protein reduction with or without amino acid supplementation is effective to improve meat quality. However, some adverse effects of low protein diets were observed at the same time, such as reduced growth performance, smaller loin area and increased backfat thickness (7, 8). Therefore, strategies need to be developed to promote IMF deposition without an increase in subcutaneous fat (improved fat partitioning) and a reduction in lean growth for pork production.

Glycine is typically deficient in low protein diets (LPD). Some studies on chicken have revealed that supplementation of glycine or its precursors to LPD overcomes the deficiency and improves animal growth performance (13, 14), which have not been conducted on pigs. Consequently, the aim of the current study was to explore the effects of supplementation of glycine to LPD on growth performance and meat quality, as well as the underlying mechanisms in growing-finishing pigs.

## 2. Materials and methods

All animal procedures were reviewed and approved by the Protocol Management and Review Committee of Institute of Subtropical Agriculture, Chinese Academy of Science (No. 8420190045).

### 2.1. Animal and dietary treatment

A total of 96 crossbred pigs (Duroc × Landrace × Yorkshire, DLY) with similar initial body weight (BW, 60.00 ± 2.00 kg) were selected and randomly assigned to 3 diet treatments (8 pens per treatment and 4 pigs per pen): (1) normal diet (ND): pigs were fed a basal diet with 16% crude protein (CP) to meet the nutritional needs for growing-finishing pigs according to the National Research Council (NRC, 2012); (2) low protein diet (LPD): pigs were fed a low protein diet with 12% CP by decreasing the content of soybean meal; (3): LPD supplemented with glycine (LPDG): pigs were fed a LPD supplemented with 0.57% glycine. All diets were isoenergetic and the chemical composition and nutrient levels of diets are listed in Supplementary Table S1. Pigs had *ad libitum* access to feed and water. The feeding trial was conducted for 43 days.

### 2.2. Sample collection

At the end of feeding trial, 8 pigs (one pig per pen) were randomly selected from each diet treatment, fasted for 12 h and slaughtered according to the regularly applied slaughter procedure at a commercial slaughter plant (Hunan New Wellful Co., LTD, Changsha, China). At 0, 0.75, 4, and 24 h postmortem (PM), a slice of *Longissimus thoracis* (LT) muscle between the 10th and 11th thoracic vertebrae in the left side of carcass was dissected, quickly cut into small pieces, mixed, and snapfrozen in liquid nitrogen for chemical, enzyme activity and metabonomics analysis. At the same time, a piece of muscle at 0 h PM was fixed for muscle fiber typing by histochemical analysis. One boneless pork loin chop was dissected at 24 h PM from the same location in the right side of carcass for sensory evaluation.

### 2.3. Chemical analysis

The percentage content (w/w) of moisture, ash, protein and lipid in muscle (0 h PM) were analyzed according to GB5009.3–2016 National Food Safety Standard-Determination of Moisture in Foods, GB5009.4–2016 National Food Safety Standard-Determination of Ash in Foods, GB5009.5–2016 National Food Safety Standard-Determination of Protein in Foods and GB5009.6–2016 National Food Safety Standard-Determination of Lipid in Foods, respectively.

### 2.4. Glycolytic assay

Glycogen, glucose and lactate in muscle were determined using commercial available assay kits (Solarbio, Beijing, China) according to the manufacturer’s instruction. Muscle glucose-6-phosphate was measured using an assay kit (Sigma, St. Louis, MO, United States). Glycolytic potential (GP) was calculated as glycolytic potential = 2 × ([glycogen] + [glucose] + [glucose-6-phosphate]) + [lactate] (15). The content of glycogen in muscle was expressed as μmol glucose/g muscle. GP was expressed as μmol lactate/g muscle. The content of glucose, glucose-6-phosphate and lactate in muscle were all expressed as μ mol/g muscle.

### 2.5. Enzyme activity analysis

The enzymatic activities of glycogen phosphorylase a (GPa), pyruvate kinase (PK), lactate dehydrogenase (LDH) and citrate synthase (CS) were determined using commercial kits (Solarbio, Beijing, China) according to the manufacturer’s procedure. Cytochrome c oxidase (CcO) activities were measured using a kit from BioVision (BioVision, Palo Alto, CA, United States). GPa activity was expressed as nanomoles of NADPH produced from glucose-6-phosphate by glucose-6-phosphate dehydrogenase per minute per milligram of muscle protein. PK activity was calculated as nanomoles of NADH oxidized per minute per milligram of muscle protein. LDH activity was expressed as nanomoles of pyruvate produced from lactate per minute per milligram of muscle protein. CS activity was calculated as nanomoles of 5′-thionitrobenzoate (TNB) produced from 5,5′-dithiobis-(2-nitrobenzoic acid) (DTNB) per minute per milligram of muscle protein. CcO activity was calculated as nanomoles of cytochrome C oxidized per minute per milligram of muscle protein.

### 2.6. Histochemical analysis of muscle fiber types

A piece of LT muscle was fixed in fixative (Servicebio, Beijing, China) for 24 h, successively dehydrated in 15 and 30% sucrose solutions at 4°C, and embedded in Tissue-Tek O.C.T. compound (Sakura, Japan). The tissue blocks were sectioned into 8–10 μm slices using a cryostat (CryoStar NX50, Thermo, United States) at −20°C.

Histochemical staining of myosin adenosine triphosphatase (m-ATPase) was performed as in literature (16). Briefly, muscle sections were first pre-incubated in solution (0.17 M Tris-base, 20 mM CaCl_2_, pH 10.4) for 5 min, then incubated in solution (0.17 M Tris-base, 18 mM CaCl_2_, 2.7 mM ATP, pH 9.4) for 30 min. Tissue sections were stained with 2% calcium chloride for 3 times with each time for 2 min, then stained with 2% cobalt nitrate for 5 min and washed, and finally stained with 1% ammonium sulfide for 30 s and washed. Tissue sections were dehydrated successively in 70, 80, 95 and 100% alcohol, diaphanized in xylene, and sealed with neutral gum.

Histological slides were scanned using a digital slide scanner (Pannoramic DESK, 3DHistech, Hungary) and muscle fibres were counted by using Image-Pro Plus 6.0 software (Media Cybernetics, Inc., United States). The percentages of type I and type II muscle fibers were calculated.

### 2.7. Sensory evaluation

Sensory evaluation was carried out by 10 panelists as in literature (17). Briefly, boneless loin chops (2 cm in thickness) were pan broiled to an internal temperature of 71°C, cut into 2 cm^3^ cubes, wrapped in pre-labeled foils and placed in a heated incubator before given to the assessors. Four samples, of which three were testing samples (one sample from each dietary treatment) and one was a reference sample (commercial pork loin chops), were given to each panelist in a session. Testing samples were scored on a 1–10 points scale in comparison to the reference sample as previously described.

### 2.8. Untargeted metabolomic analysis

Muscle tissues were pulverized in liquid nitrogen. 100 mg of powdered sample were suspended in 500 μl of prechilled 80% methanol containing 0.1% formic acid and homogenized for metabolite extraction. The samples were centrifuged at 9000 rpm, 4°C for 5 min. The supernatants were collected and diluted with LC–MS grade water to a concentration containing 60% methanol. The extracts were passed through a 0.22 μm filter and used for LC–MS/MS analysis.

LC–MS/MS analyses were performed using a Vanquish UHPLC system (Thermo Fisher) coupled with an Orbitrap Q Exactive HF-X mass spectrometer (Thermo Fisher). Samples were injected onto a Hyperil Gold column (1.9 μm,100 × 2.1 mm, Thermo Fisher) and eluted using a 16-min linear gradient at a flow rate of 0.2 ml/min. The mobile phases for the positive ion mode were 0.1% formic acid in water (A) and methanol (B). The mobile phases for the negative ion mode were 5 mM ammonium acetate (pH 9.0) (A) and methanol (B). The elution gradient was as follows: 0 min, 2% B, 1.5 min, 2% B; 12 min, 100% B; 14.0 min, 100% B; 14.1 min, 2% B; 16 min, 2% B. Q Exactive HF-X mass spectrometer was operated in positive/negative polarity mode with spray voltage 3.2 kV, capillary temperature 320°C, sheath gas flow rate 35 arb and aux gas flow rate 10 arb.

The raw data generated by UHPLC–MS/MS were processed using the Compound Discoverer (version 3.0, Thermo Fisher) to perform peak alignment, peak picking, and quantification of each metabolite. The main parameters were set as follows: retention time tolerance = 0.2 min, mass tolerance = 5 ppm; signal intensity tolerance = 30%; signal/noise ratio = 3; minimum intensity = 100,000. Peak intensities were normalized to the total spectral intensity. The normalized data was used to predict the molecular formula based on additive ions, molecular ion peaks and fragment ions. Peaks were matched to the mzCloud^2^ and ChemSpider^3^ database for peak annotation and quantification.

### 2.9. Statistical analysis

Data analysis was performed by using SPSS 25.0 (SPSS Inc., Chicago, IL, United States). One-way ANOVA was conducted using the mean values measurements followed by Fisher’s protected least significant difference test, with individual pig (*n* = 8) as the experimental unit. Sensory evaluation data were analyzed using a mixed model procedure where diet treatments, sessions, and their interactions were treated as fixed effects and panelist as random effect. Levels for significant differences were set at *p* < 0.05. Mean values and standard errors of the means were reported. Principle component analysis (PCA) was performed to explore the relationships between sensory quality traits and total lipid content among the dietary treatments. The data were normalized by setting the mean values to zero and scaling on the basis of one standard deviation. The graphs were plotted using OriginPro 8 software.

## 3. Results and discussion

### 3.1. Low protein diet supplemented with glycine increased IMF but decreased glycogen in muscle

Many studies have reported that reduced protein diets increase body fat accumulation, IMF content and marbling of growing-finishing pigs (1), showing that protein restriction could be used to improvemeat quality. However, this is usually accompanied by reduced animal growth rate and lean meat yield (7, 8). For this reason, the effect of LPD supplemented with glycine on pig growth, muscle metabolism and meat quality were studied. The growth performance of pigs is shown in Supplementary Table S2. LPD decreased animal average daily weight gain (ADG) from 855 ± 17 g to 777 ± 15 g (*p* < 0.05) when compared to that of ND, but supplementation of glycine to LPD increased ADG to 839 ± 31 g, which was not different (*p* > 0.05) to that of the ND treatment, showing that dietary supplementation of glycine to LPD eliminated the negative effect of protein restriction on animal growth performance.

As shown in Table 1, LPD and LPDG had no effect on moisture, protein and ash content in porcine muscle, but LPDG significantly increased (*p* < 0.05) the deposition of fat within muscle, which is about 60% higher than that of control ND pigs. Although it is not statistically significant, low protein diet without glycine supplementation increased IMF from 1.83 ± 0.23 to 2.64 ± 0.27% when compared to the control normal diet. This is in agreement with literature that LPD combined with or without amino acid supplementation increase pig fat content (1). As IMF is a key factor determining meat quality (3), the increased IMF content in muscle of LPDG pigs should improve meat quality. At the same time, both glycogen content and glycolytic potential were significantly lower in the two low protein diets with or without glycine supplementation when compared to the control normal diet. As glycogen is the substrate of postmortem glycolysis, which plays a key role in determining muscle ultimate pH values and in the postmortem formation of meat quality, the reduced glycogen content in pig muscle indicates that low protein diet influences pork meat quality by altering muscle carbonhydrate metabolism. Taken together, these data show that LPD and LPDG altered nutrient metabolism in the muscle of growing-finishing pigs, with decreased glycogen accumulation but increased fat deposition. LPD impaired animal growth performance, while supplementation of glycine to LPD recovered pig growth rate.

**Table 1:** Chemical composition of LT muscle from growing-finishing pigs receiving a normal, low protein, or low protein supplemented with glycine diet.

	ND	LPD	LPDG
Moisture, %	73.57 ± 0.56	70.91 ± 0.93	71.57 ± 1.51
Ash, %	1.31 ± 0.04	1.38 ± 0.04	1.42 ± 0.11
Proteins, %	23.99 ± 0.36	23.93 ± 0.37	24.42 ± 0.46
Fat, %	1.83 ± 0.23^b^	2.64 ± 0.27^ab^	2.92 ± 0.38^a^
Glycogen, μmol glucose/g muscle	18.68 ± 1.71^a^	11.81 ± 2.41^b^	12.42 ± 1.95^b^
Glucose, μmol /g muscle	4.80 ± 0.32	4.74 ± 0.37	4.58 ± 0.26
G-6-P, μmol /g muscle	4.84 ± 0.09^b^	5.51 ± 0.07^a^	4.99 ± 0.14^b^
Lactate, μmol /g muscle	64.85 ± 2.43	59.26 ± 3.67	59.62 ± 2.66
GP, μmol lactate/g muscle	121.49 ± 3.66^a^	103.38 ± 6.11^b^	103.60 ± 3.98^b^

G-6-P, glucose-6-phosphate; GP, glycolytic potential.

ab Within a row, means lacking a common letter are significantly different at *p* < 0.05. *n* = 8.

### 3.2. Low protein diet supplemented with glycine improved meat quality

The sensory data are presented in Table 2. In consistence with IMF content, LPDG significantly increased pork loin tenderness, juiciness and the overall liking score when compared with ND. This is reasonable as IMF greatly affect meat tenderness, juiciness and overall acceptability, which are negatively affected when IMF < 2–2.5% (3). Both LPD and LPDG increased pork IMF from 1.83 to over 2.5%, demonstrating that LPD with or without glycine supplementation had a positive effect on meat quality. Indeed, LPD without glycine supplementation also increased meat tenderness and overall liking score though juiciness of pork was not affected (Table 2). As lipids are major source of flavor compounds in pork (18), especially volatile flavor compounds, like aldehydes, ketones and alcohols which are commonly formed by fat oxidation or thermal degradation (17, 19), it is reasonable to observed that LPD and LPDG enhanced the meaty odor of pork loin (Table 2).

**Table 2:** Sensory panel evaluation of pork loin from growing-finishing pigs receiving a normal, low protein, or low protein supplemented with glycine diet.

Items	Dietary treatments				Significance	
	ND	LPD	LPDG	Diet	Session	Diet × session
Tenderness	5.93 ± 0.08^c^	6.54 ± 0.08^b^	7.11 ± 0.10^a^	^***^	n.s.	n.s.
Juiciness	6.12 ± 0.09^b^	6.32 ± 0.06^b^	6.66 ± 0.10^a^	^**^	n.s.	n.s.
Umami	6.29 ± 0.07^c^	6.51 ± 0.07^b^	6.91 ± 0.09^a^	^**^	n.s.	n.s.
Sweetness	6.17 ± 0.09	6.28 ± 0.06	6.22 ± 0.09	n.s.	n.s.	n.s.
Unpleasant taste	5.75 ± 0.12^a^	5.09 ± 0.12^b^	4.9 ± 0.14^b^	^**^	n.s.	n.s.
Meaty odor	6.26 ± 0.08^b^	6.56 ± 0.06^a^	6.57 ± 0.10^a^	^**^	n.s.	n.s.
Unpleasant odor	5.30 ± 0.14	5.04 ± 0.13	5.11 ± 0.12	n.s.	n.s.	n.s.
Overall liking	6.44 ± 0.07^c^	6.71 ± 0.07^b^	7.04 ± 0.09^a^	^***^	n.s.	n.s.

^abc^Within a row, means without a common letter are significantly different at *p* < 0.05. *n* = 8. n.s.: not significant. ^**^p < 0.01; ^***^p < 0.001.

To better show the impact of diets on meat quality, principle component analysis was performed to explore the relationships between sensoryquality traits, total lipid content and the dietary treatments. As shown in Supplementary Figure S1, PC1 explained 65.30 of the variance associated with meat quality traits and could represent the overall quality of meat. PC1 separated LPDG and LPD from ND treatments, showing meat of the two low protein diet treatments had higher quality than meat of the ND treatment. In addition, the lipid content, meat juiciness, tenderness and overall liking are all in the first quadrant, showing the close relationship between meat lipid content and these meat quality traits. In summary, these data show that IMF had an important impact on meat quality. LPDG improved meat quality by increasing IMF content.

### 3.3. Low protein diet supplemented with glycine altered metabolic enzyme activities and muscle fiber typing

**Figure 1:**
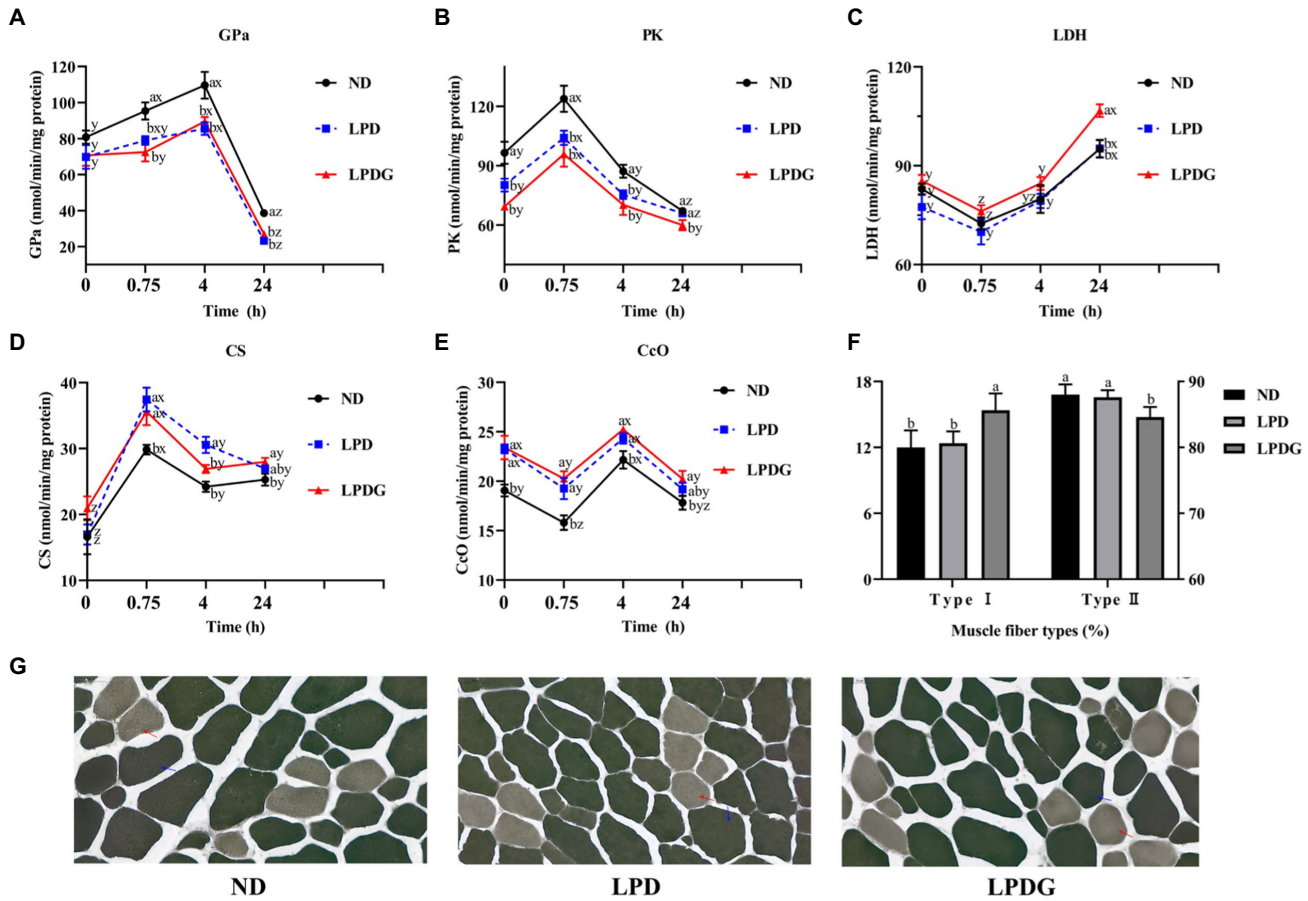
Metabolic enzyme activities and muscle fiber type composition of LT muscle from pigs receiving a normal, low protein, or low protein supplemented with glycine diet. **(A**-**E)**: glycogen phosphorylase a (GPa), pyruvate kinase (PK), lactate dehydrogenase (LDH), citrate synthase (CS) and cytochrome c oxidase (CcO) activities determined in LT muscle within 24  h PM. **(F)**: the percentages of type I and type II muscle fibres within LT muscle. **(G)**: histochemical staining of m-ATPase. LPDG increased the percentage of type I (red arrow), but decreased the number of type II muscle fibers. ^ab^At the same PM time point, means lacking a common letter differ at *p* < 0.05. ^xyz^Within the same dietary treatment, means lacking a common letter differ at *p* < 0.05. *n* = 8.

To verify and better understand the altered metabolism within the muscle, the activities of some glycogenolytic/glycolytic and oxidative enzymes in LT muscle were measured. As shown in Figure 1, the activities of PK at 0 h PM were significantly lower, but the activities of CcO were significantly higher (*p* < 0.05) for the LPD and LPDG treatments compared to ND treatment, which represent the *in vivo* enzyme activities. This shows that protein reduction in diets increased animal muscle oxidative metabolism capacity, but decreased glycolytic capacity. In addition, higher activities of GPa and PK at both 0.75 and 4 h PM, and lower activities of CS at 0.75 h PM, as well as lower activities of CcO at 0.75 and 4 h PM were determined for ND pigs compared to LPD and LPDG pigs. These data further show that protein reduction in diets increased animal muscle oxidative capacity, but decreased glycolytic ability. LDH activity was similar among treatments till 4 h PM. This is logic because LDH is not a rate-limiting enzyme and its activity may not correlate with muscle glycolytic/oxidative capacity. The difference in LDH activity among dietary treatments at 24 h PM may reflect difference in the stability of this protein.

According to the m-ATPase activity, muscle fibers can be classified into type I and type II muscle fibers (20, 21). Type I muscle fiber is also known as slow contraction muscle fiber, oxidative muscle fiber or red muscle fiber. Type I fibers are rich in mitochondria and lipids, have high aerobic metabolic enzymes activity (such as cytochrome oxidase, succinate dehydrogenase, citrate synthase, etc.) and low ATPase activity. In addition, slow muscles are rich in capillaries and myoglobin, have strong oxygen carrying capacity, twitch slowly and lastingly. Type II muscle fibers are fast contraction muscle fibers, glycolytic muscle fibers or white muscle fibers. Type II fibers are rich in glycogen, have high glycolytic enzyme activities and m-ATPase activity, twitch fast but not lastingly. Histochemical analysis of m-ATPase activity revealed that LPDG increased the relative number of type I muscle fiber and simultaneously decreased the number of type II muscle fiber within LT muscle (Figures 1F,G), showing that LPDG promoted the transition of porcine muscle fiber from type II to type I. Type I myofiber usually have smaller diameter and higher lipid content, it is reasonable that the meat of LPDG group was more tender and better in flavor (Table 2). Several studies have reported that porcine muscles with more type I muscle fibers have up-regulated oxidative metabolism enzymes, lower glycolytic potential, reduced postmortem glycolysis and higher meat quality (22–24). Based on the literature and the data obtained in the present study, it can be concluded that LPDG promoted the transition of muscle fibers from type II to type I, altered muscle energy metabolism, increased IMF content and thus improved meat quality.

### 3.4. Low protein diet supplemented with or without glycine altered metabolome in porcine muscle

For better exploration of the changes induced by diets, metabolic profiling of LT muscle was performed using UHPLC–MS/MS, which identified 859 metabolites in positive mode and 805 metabolites in negative mode. As 156 metabolites were identified in both positive and negative modes, a total of 1,508 metabolites were identified and applied for MetaboAnalyst analysis in the present study.

**Figure 2:**
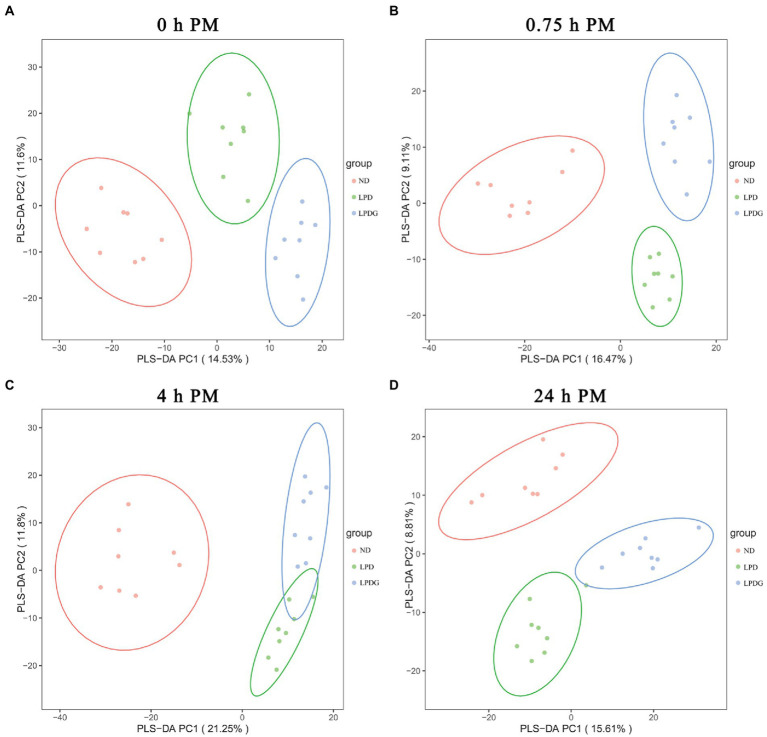
The PLS-DA score plot of metabolites in LT muscle from pigs receiving anormal, low protein, or low protein supplemented with glycine diet.

To provide an overview of the difference in metabolic profiles, partial least squares discrimination analysis (PLS-DA) was conducted. As shown in Figure 2, PLS-DA effectively and distinctly separated LT muscle samples from each cohort based on animal diets, showing that LPD and LPDG caused a significant change in the overall metabolite profile of the LT muscle within 24 h PM. In other words, pigs receiving a normal, a low protein diet or a low protein diet supplemented with glycine had distinct metabolic patterns in postmortem skeletal muscle. As the metabolite profile determined in muscle collected at 0 h PM represents the *in vivo* metabolism, the good segregation of ND and LPDG muscle samples with LPD samples positioned in between these two cohorts (Figure 2A) suggests that supplementation of glycine had additive effects to low protein diet and further altered the metabolism in skeletal muscle of pigs. In addition, the ND muscle samples located relatively further away from the two low protein diet groups (Figure 2), suggesting that low protein diet caused greater changes in metabolism than glycine supplementation did. This is in agreement with glycogen content (Table 1) and the activities of PK and CcO at 0 h PM (Figure 1), all of which were significantly higher or lower (*p* < 0.05) in the ND group, but not different between the two low protein diets, suggesting glycogen content, PK and CcO activities could be good indicators of muscle metabolism. To provide an overview of differentially abundant metabolites in the three dietary groups, hierarchical clustering analysis (HCA) was performed using all the annotated significantly differentially expressed metabolites and results are shown in a heatmap (Supplementary Figure S2). HCA also showed that porcine muscle samples at 0 h PM were separated according to dietary treatments, with all the LPDG samples clustering on the left side, most of ND samples (6/8) clustering on the right side, and LPD samples being intermixed with one ND samples in the middle of the panel (Supplementary Figure S2A). Once again, this profiling pattern revealed that low protein diet obviously changed metabolism in living animals and the supplementation of glycine to LPD enlarged the gap. Additionally, metabolite profiling well clustered the eight ND samples together, but LPD samples were intermixed with LPDG samples collected at 0.75 and 4 h PM (Supplementary Figure S2B,C), revealing that low protein diet caused greater postmortem metabolic changes in comparison to normal diet than glycine supplementation did in comparison to low protein diet.

The differential metabolites between diet groups were filtered according to the following statistical significance criteria: thresholds with VIP > 1, *p* < 0.05 and FC > 1.5 or FC < 0.667 and visually summarized in volcano plots (Supplementary Figure S3). The metabolites that contributed to the metabolomic difference in pairwise comparisons were marked with the circles size and color-coded based on VIP and *p* values from the PLS-DA analysis. The detailed information of these differential metabolites from LT muscle at 0, 0.75, 4, and 24 h PM are listed in Table 3 and Supplementary Table S1. Most of the differential metabolites are carbohydrate, amino acids and their derivatives, lipids, purine and cofactors.

**Table 3:** Significantly altered metabolites in LT muscle (collected at 0 h PM) from pigs fed a low protein diet in comparison to pigs fed the normal diet, and pigs fed a low protein diet supplemented with glycine in comparison to pigs fed the low protein diet.

Metabolites	KEGG ID	LPD *VS* ND				LPDG *VS* LPD			
		0 h PM				0 h PM			
		FC	VIP	*p*	Trend	FC	VIP	*p*	Trend
*Amino acids and their derivatives*									
Phenylacetylglycine	C05598	0.653	1.041	0.004	↓				
L-tyrosine	C00082	0.494	1.728	0.001	↓	1.699	1.423	0.002	↑
N-acetylleucine	C02710	0.642	1.149	0.007	↓				
N-acetylornithine	C00437	0.665	1.043	0.029	↓	0.615	1.679	0.044	↓
N-acetyl-L-aspartic acid	C01042					1.676	1.373	0.046	↑
L-glutamic acid	C00025					1.644	1.272	0.001	↑
Reduced glutathione	C00051					1.723	1.389	0.001	↑
Spermidine	C00315					1.559	1.123	0.000	↑
*Purine and cofactors*									
NMN	C00455	5.171	3.257	0.003	↑				
Adenosine tetraphosphate	C03483	6.858	3.240	0.021	↑				
Pantothenic acid	C00864					1.676	1.226	0.001	↑
Niacin	C00253					1.910	1.430	0.035	↑
NADH	C00004					2.258	1.908	0.047	↑
*Lipid*									
Acetyl-CoA	C00024	0.379	2.481	0.010	↓				
Erucic acid	C08316	2.141	4.039	0.005	↑				
Linoleic acid	C01595	2.138	2.201	0.005	↑				
ω-9-hydroperoxyarachidonic acid		1.788	1.416	000	↑				
15S-hydroxyeicosatrienoic acid		1.602	1.279	0.014	↑				
O-arachidonoyl ethanolamine		1.824	1.405	0.007	↑				
Linoleyl carnitine	6.606	4.921	0.001	↑				
O-oleoylcarnitine	3.127	2.921	0.017	↑				
(2E)-hexadecenoylcarnitine	3.622	3.481	0.017	↑				
3-hydroxydodecanoylcarnitine		2.586	2.220	0.004	↑				
3, 5-tetradecadiencarnitine	3.354	2.643	0.049	↑				
3-hydroxyhexadecadienoylcarnitine		3.513	2.338	0.037	↑				
Traumatic acid	C16308					1.730	1.266	0.006	↑
Eicosapentaenoic acid	C06428					0.664	1.089	0.045	↓
Ursolic acid	C08988					0.377	2.790	0.004	↓
Docosahexaenoic acid	C06429					0.474	1.796	0.004	↓
Arachidonic acid	C00219					0.587	1.237	0.037	↓

In accordance with previous researchers reported that diets influence protein/amino acid metabolism (25–27), low protein diet reduced the content of multiple amino acids in porcine LT muscle when compared to the normal diet (Table 3), including L-tyrosine, phenylacetylglycine, N-acetylleucine, and N-acetylornithine. However, supplementation of glycine to LPD reversed the content of L-tyrosine. In addition, glycine supplementation increased the concentrations of L-glutamic acid and N-acetyl-L-aspartic acid, as well as reduced glutathione and spermidine. Based on these data and pig growth performance, it may be speculated that LPD induced insufficiency of amino acids, especially tyrosine, could be responsible for decreased muscle protein deposition and thus the decreased animal ADG (28). Supplementation of glycine may have promoted the synthesis of and saved essential amino acids (EAA) for nonessential amino acids (NEAA), promoting muscle protein synthesis and energy metabolism (29). N-acetylornithine was decreased in both low protein diet groups when compared to the normal diet.

In agreement to the increased concentration of reduced glutathione, glycine increased the concentrations of NADH and its precursor niacin (Table 3), showing elevated antioxidant potential and reflecting altered metabolism of muscle from LPDG pigs. Pantothenic acid is a precursor of coenzyme A (CoA) and a structural element of acyl carrier protein, participating in numerous cellular catabolic and synthetic reactions. Severe deficiency of pantothenic acid in rats causes weight loss, alterations in the fur coat and finally death (30). Previous study reports that dietary pantothenic acid increases nitrogen retention efficiency, growth rate and final body weight of fish (31). In the present study, supplementation of glycine to LPD increased pantothenic acid content in porcine muscle, which should have contributed to the recovered growth performance of LPDG pigs.

Lipid metabolism is greatly altered by protein restriction. As shown in Table 3, multiple fatty acids and derivatives, including erucic acid, linoleic acid, ω-9-hydroperoxyarachidonic acid, 15 s-hydroxyeicosatrienoic acid, and O-arachidonoyl ethanolamine were significantly increased in both low protein diet groups when compared to the ND group. In addition, multiple acylcarnitines of different chain-length specificities were determined to be significantly increased by low protein diets, which include linoleyl carnitine, O-oleoylcarnitine, (2E)-hexadecenoylcarnitine, 3-hydroxydodecanoylcarnitine, 3, 5-tetradecadiencarnitine, and 3-hydroxyhexadecadienoylcarnitine. These data revealed that low protein diets promoted not only IMF deposition as previously reported (1), but fatty acid transport across the mitochondrial membrane and oxidation. As slow muscle fibers are more dependent on fatty acid catabolism and oxidative phosphorylation for ATP production, the increased fat mobilization in the muscle of pigs fed low protein diets suggested improved oxidative capacity as revealed by the increased enzymatic activities of CS and CcO (Figures 1D,E). This is especially true for the LPDG pigs as glycine supplementation significantly increased the percentage of slow type (type I) muscle fiber in porcine LT muscle (Figures 1F,G). When the two low protein diet groups were compared, glycine supplementation decreased muscle content of some polyunsaturated fatty acids, including eicosapentaenoic acid, docosahexaenoic acid, and arachidonic acid, which is likely related to the increased pantothenic acid content of LPDG group as pantothenic acid alters the accumulation of saturated and unsaturated fatty acids in fish liver and muscle (31). Acetyl-CoA is an important intermediate metabolite of energy metabolism. Carbohydrate, fatty acid and amino acids converged on acetyl-CoA before entering into tricarboxylic cycle for complete oxidation and ATP generation through phosphorylation. Acetyl-CoA is also the substrate for gluconeogenesis, ketogenesis and fatty acid synthesis. The reduced acetyl-CoA in the two low protein diet groups revealed a comprehensive alteration in muscle metabolism induced by dietary protein restriction.

Diets also induced metabolism alteration in postmortem muscle. Some differentially expressed metabolites, which may be related to the conversion of muscle to meat, are listed in Supplementary Table S3. Glycolysis determined the rate of muscle pH decline and ultimate muscle pH values. In the present study, three intermediates of glycolysis, glucose 6-phosphate, glyceraldehyde 3-phosphate and 3-phosphoglyceric acid were detected to be differential in abundance between the ND and the two low protein diet groups at 0.75 PM. Another feature of postmortem muscle is that many amino acids and peptides were increased in abundance in postmortem muscle by protein restriction, of which some were detected to be lower in the LPDG group at 24 h PM when the two low protein diet groups were compared, indicating that low protein diets promoted proteolysis, which influence the aging of meat and final meat quality, such as meat tenderness and taste. Higher concentrations of AMP, ADP and UMP were also determined in the two low protein diets. In summary, all these data showed that dietary protein restriction and glycine supplementation influenced glycolysis, proteolysis and nucleotide metabolism in postmortem muscle and likely the conversion of muscle to meat and meat quality.

**Figure 3:**
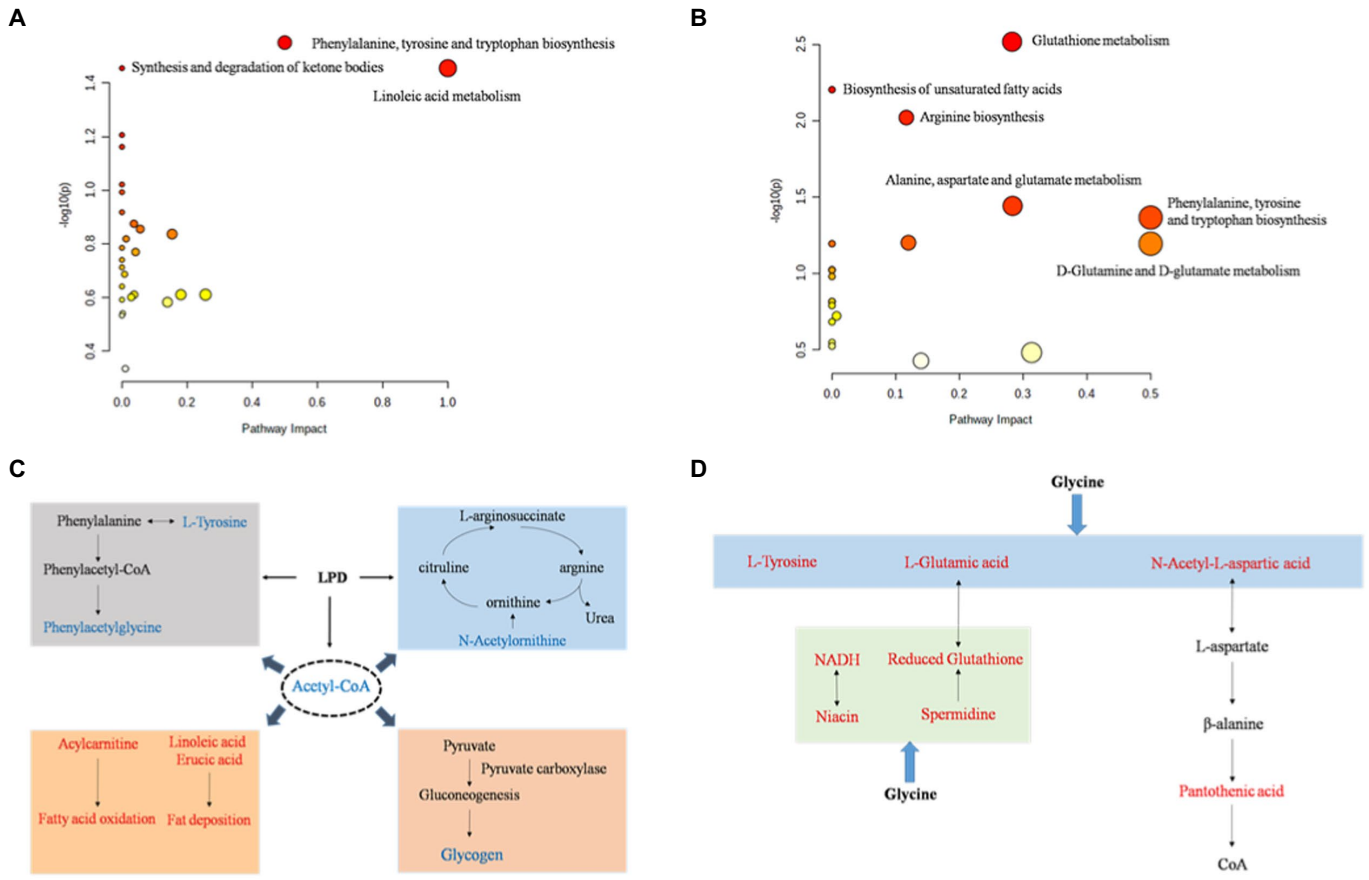
The perturbed metabolic pathways **(A**, **B)** and networks **(C**, **D)** associated with low protein diet **(A**, **C)** and glycine supplementation to low protein diet **(B**, **D)**. Metabolites in red were up-regulated and metabolites in blue were down-regulated. *p* < 0.05, *n* = 8.

To further assess the biochemical perturbations at the stage of pig growth induced by LPD and LPDG, pathway enrichment analysis was performed based on the identified metabolites that were significantly differentially expressed. The KEGG enrichment analysis (Supplementary Tables S4, S5) showed that 27 and 20 metabolism pathways including amino acid metabolism pathways, lipid metabolism pathways, carbohydrate metabolism pathways, and nucleotide metabolism pathways were predicted according to identified metabolites in LPD and LPDG groups, respectively. According to the metabolic pathway analysis results from the MetaboAnalyst 4.0 program (Figure 3, raw data with a *p* < 0.05), phenylalanine, tyrosine and tryptophan biosynthesis, synthesis and degradation of ketone bodies, and linoleic acid metabolism were significantly affected by LPD (Figure 3A). Meanwhile, D-glutamine and D-glutamate metabolism, glutathione metabolism, biosynthesis of unsaturated fatty acids, arginine biosynthesis, alanine, aspartate and glutamate metabolism, phenylalanine, tyrosine and tryptophan biosynthesis were significantly altered by LPDG (Figure 3B).

Based on the KEGG database, the main pathways contributing to the metabolite differences of pig LT muscle by LPD and LPDG were summarized and sketched in Figures 3C,D. Traditionally, amino acids are classified as EAA and NEAA. However, growing literatures report that some of the traditionally classified NEAA play important regulatory roles in cell signaling, gene expression, protein turnover, nutrient metabolism, and oxidative defense (32). In the present study, LPD impaired animal growth and significantly reduced the ADG of growing-finishing pigs (28). At the same time, multiple amino acids in LT muscle of LPD pigs decreased in abundance, including L-tyrosine, phenylacetylglycine, N-acetylleucine, and N-acetylornithine (Table 3). Although the physiological regulatory function of these amino acids are not well understood, it is likely that protein restriction of LPD group led to insufficiency of tyrosine and phenylacetylglycine, and thus reduced protein synthesis and animal growth (Figure 3C). N-acetylornithine was decreased in both low protein diet groups when compared to the normal diet. As N-acetylornithine is deacetylated to produce ornithine, the decreased N-acetylornithine could be a mechanism for reduced nitrogen emission through urea cycle induced by low protein diets (Figure 3C). Acetyl-CoA was significantly reduced in both low protein diet groups, which could at least partially be an accumulated effect of fatty acid synthesis and oxidation induced by protein restriction (Figure 3C). On the other hand, reduced acetyl-CoA by low protein diets may regulate intracellular metabolism. Previous studies reveal that acetyl-CoA modulates the activity of pyruvate carboxylase (PC) and decreased acetyl-CoA in liver suppresses hepatic glucose production (33). It is likely that low protein diet induced fatty acid mobilization reduced acetyl-CoA in porcine muscle and thus likely liver, which then suppressed PC activity, hepatic glucose production and glycogen accumulation in the muscle of animals (Figure 3C).

Glycine supplementation to low protein diet reversed L-tyrosine in porcine muscle to the same level as of ND group (Table 3). In addition, glycine supplement significantly increased muscle content of L-glutamic acid and N-acetyl-L-aspartic acid, which can be used for L-aspartic acid and pantothenic acid synthesis. These data revealed that glycine supplementation to LPD could promote the synthesis of NEAA such as tyrosine, glutamic acid and aspartic acid, and pantothenic acid, the precursor of coenzyme A and acyl carrier protein, to promote protein accretion and animal growth (Figure 3D).

## 4. Conclusion

Protein restriction to growing-finishing pigs improved intramuscular fat deposition, the activities of glycolytic enzymes GPa and PK and meat quality, but decreased glycogen content and the activities of oxidative metabolic enzymes CS and CcO in porcine skeletal muscle when compared with normal diets. Supplementation of glycine to low protein diet promoted muscle fiber transition from type II to type I, and further improved pork tenderness and overall sensory quality. Metabolic analysis revealed that diets affected not only nutrients and energy metabolism *in vivo*, but also energy metabolism and proteolysis in postmortem muscle. Pathway analysis suggested that low protein diets likely caused insufficiency of tyrosine and its metabolites, which could lead to decreased muscle protein deposition and impaired animal growth performance, and reduced nitrogen emission through urea cycle. Low protein diets also decreased the abundance of acetyl-CoA in muscle, which may be involved in the increased lipid mobilization and down-regulate glycogen accumulation in muscle. Supplementation of glycine to low protein diets increased the muscle content of tyrosine, glutamic acid and N-acetyl-aspartic acid, which could promote the synthesis of NEAA and pantothenic acid, save EAA for NEAA synthesis, and thus promote protein accretion and animal growth. Further studies are necessary to understand the detailed mechanism of glycine induced myofiber transition and improved animal growth performance, which are directly related to meat production efficiency.

## Data availability statement

The data presented in the study are deposited in the Metabolights repository, accession number MTBLS7345. https://www.ebi.ac.uk/metabolights/MTBLS7345.

## Ethics statement

The animal study was reviewed and approved by Protocol Management and Review Committee of Institute of Subtropical Agriculture, Chinese Academy of Science.

## Author contributions

SJ: data curation, formal analysis, and investigation. WQ: software and methodology. JL: formal analysis and formal analysis. AL: methodology. XZ: resources. FL: supervision and validation. QS: funding acquisition, conceptualizat, and writing—original draft. No conflicts of interest are declared for any of the authors. All authors contributed to the article and approved the submitted version.

## Funding

This study was supported by the National Key R&D Program of China [Grant no. 2018YFD0500405] and National Top Disciplines Development Project for Innovation Teams [Grant no. kxk201801004].

## Conflict of interest

The authors declare that the research was conducted in the absence of any commercial or financial relationships that could be construed as a potential conflict of interest.

## Publisher’s note

All claims expressed in this article are solely those of the authors and do not necessarily represent those of their affiliated organizations, or those of the publisher, the editors and the reviewers. Any product that may be evaluated in this article, or claim that may be made by its manufacturer, is not guaranteed or endorsed by the publisher.

## Footnotes

^1^ http://www.stats.gov.cn/xxgk/sjfb/tjgb2020/201902/t20190228_1768642.html

^2^ https://www.mzcloud.org/

^3^ http://www.chemspider.com/

## References

[1] Alfaia C. M. Lopes P. A. Madeira M. S. Pestana J. M. Coelho D. Toldrá F.. (2019), 'Current feeding strategies to improve pork intramuscular fat content and its nutritional quality'. Adv. Food Nutr. Res., 89, 53–94, doi: 10.1016/bs.afnr.2019.03.006, PMID: . 31351530

[2] Hocquette J. F. Gondret F. Baéza E. Médale F. Jurie C. Pethick D. W. (2010), 'Intramuscular fat content in meat-producing animals: development, genetic and nutritional control, and identification of putative markers'. Animal, 4:303–319, doi: 10.1017/S1751731109991091, PMID: 22443885

[3] DeVol D. L. McKeith F. K. Bechtel P. J. Novakofski J. Shanks R. D. Carr T. R. (1988), 'Variation in composition and palatability traits and relationships between muscle characteristics and palatability in a random sample of pork Carcasses1'. J. Anim. Sci., 66, 385–395. doi: 10.2527/jas1988.662385x

[4] Daza A. Mateos A. Rey A. I. Ovejero I. López-Bote C. J. (2007), 'Effect of duration of feeding under free-range conditions on production results and carcass and fat quality in Iberian pigs'. Meat Sci., 76, 411–416, doi: 10.1016/j.meatsci.2006.10.004, PMID: 22060982

[5] Madeira M. S. Pires V. M. R. Alfaia C. M. Costa A. S. H. Luxton R. Doran O.. (2013), 'Differential effects of reduced protein diets on fatty acid composition and gene expression in muscle and subcutaneous adipose tissue of Alentejana purebred and large white x landrace x Pietrain crossbred pigs. Br. J. Nutr., 110, 216–229, doi: 10.1017/S0007114512004916, PMID: 23286604

[6] Castell A. G. Cliplef R. L. Poste-Flynn L. M. Butler G. (1994), 'Performance, carcass and pork characteristics of castrates and gilts self-fed diets differing in protein content and lysine:energy ratio'. Can. J. Anim. Sci., 74, 519–528, doi: 10.4141/cjas94-073

[7] Goerl K. F. Eilert S. J. Mandigo R. W. Chen H. Y. Miller P. S. (1995), 'Pork characteristics as affected by two populations of swine and six crude protein levels'. J. Anim. Sci., 73, 3621–3626. doi: 10.2527/1995.73123621x 8655436

[8] Kerr B. J. McKeith F. K. Easter R. A. (1995), 'Effect on performance and carcass characteristics of nursery to finisher pigs fed reduced crude protein, amino acid-supplemented diets', J. Anim. Sci., 73, 433–440, doi: 10.2527/1995.732433x, PMID: 7601776

[9] Ma X. Zheng C. Hu Y. Wang L. Yang X. Jiang Z. (2015), 'Dietary L-arginine supplementation affects the skeletal Longissimus muscle proteome in finishing pigs'. PLoS One, 10:e0117294. doi: 10.1371/journal.pone.0117294 25635834 PMC4311982

[10] Tan B. Yin Y. Liu Z. Li X. Xu H. Kong X.. (2009), 'Dietary l-arginine supplementation increases muscle gain and reduces body fat mass in growing-finishing pigs', Amino Acids, 37, 169–175, doi: 10.1007/s00726-008-0148-0, PMID: 18683021

[11] Madeira M. S. Alfaia C. M. Costa P. Lopes P. A. Lemos J. P. C. Bessa R. J. B. (2014), 'The combination of arginine and leucine supplementation of reduced crude protein diets for boars increases eating quality of pork', J. Anim. Sci., 92, 2030–2040, doi: 10.2527/jas.2013-6876 24663178

[12] Tous N. Lizardo R. Vilà B. Gispert M. Font-i-Furnols M. Esteve-Garcia E. (2016), 'Addition of arginine and leucine to low or normal protein diets: performance, carcass characteristics and intramuscular fat of finishing pigs', Span. J. Agric. Res., 14, doi: 10.5424/sjar/2016144-9351

[13] Dean D. Bidner T. D. Southern L. L. (2006), 'Glycine supplementation to low protein, amino acid-supplemented diets supports optimal performance of broiler chicks', Poult. Sci., 85, 288–296, doi: 10.1093/ps/85.2.288. 16523629

[14] Ospina-Rojas I. C. Murakami A. E. Oliveira C. A. L. Guerra A. F. Q. G. (2013), 'Supplemental glycine and threonine effects on performance, intestinal mucosa development, and nutrient utilization of growing broiler chickens', Poult. Sci., 92, 2724–2731, doi: 10.3382/ps.2013-03171, PMID: 24046420

[15] Monin G. Sellier P. (1985), 'Pork of low technological quality with a normal rate of muscle pH fall in the immediate post-mortem period: the case of the Hampshire breed', Meat Sci., 13, 49–63, doi: 10.1016/S0309-1740(85)80004-8, PMID: 22055445

[16] Hwang Y. H. Kim G. D. Jeong J. Y. Hur S. J. Joo S. T. (2010), 'The relationship between muscle fiber characteristics and meat quality traits of highly marbled Hanwoo (Korean native cattle) steers', Meat Sci., 86, 456–461, doi: 10.1016/j.meatsci.2010.05.034 20598446

[17] Guo Q. Kong X. Hu C. Zhou B. Wang C. Shen Q. W. (2019), 'Fatty acid content, flavor compounds, and sensory quality of pork loin as affected by dietary supplementation with l-arginine and glutamic acid', J. Food Sci., 84, 3445–3453, doi: 10.1111/1750-3841.14959 31762038

[18] Scollan N. D. Dannenberger D. Nuernberg K. Richardson I. MacKintosh S. Hocquette J. F.. (2014), 'Enhancing the nutritional and health value of beef lipids and their relationship with meat quality', Meat Sci., 97:384–394, doi: 10.1016/j.meatsci.2014.02.015, PMID: 24697921

[19] Calkins C. R. Hodgen J. M. (2007), 'A fresh look at meat flavor', Meat Sci., 77, 63–80, doi: 10.1016/j.meatsci.2007.04.016 22061397

[20] Brooke M. H. Kaiser K. K. (1970), 'Muscle fiber types: how many and what kind?', Arch. Neurol., 23, 369–379, doi: 10.1001/archneur.1970.00480280083010, PMID: 4248905

[21] Lind A. Kernell D. (1991), 'Myofibrillar ATPase histochemistry of rat skeletal muscles: a "two-dimensional" quantitative approach', J. Histochem. Cytochem., 39, 589–597, doi: 10.1177/39.5.1826695, PMID: . 1826695

[22] Kim G. D. Yang H. S. Jeong J. Y. (2018), 'Intramuscular variations of proteome and muscle fiber type distribution in semimembranosus and semitendinosus muscles associated with pork quality', Food Chem., 244, 143–152, doi: 10.1016/j.foodchem.2017.10.046, PMID: 29120762

[23] Li Y. Li J. Zhang L. Yu C. Lin M. Gao F.. (2015), 'Effects of dietary energy sources on post mortem glycolysis, meat quality and muscle fibre type transformation of finishing pigs', PLoS One, 10,:e0131958, doi: 10.1371/journal.pone.0131958, PMID: 26125946 PMC4488424

[24] Zhang S. H. Zhu L. Wu Z. H. Zhang Y. Tang G. Q. Jiang Y. Z.. (2013), 'Effect of muscle-fiber type on glycogenin-1 gene expression and its relationship with the glycolytic potential and pH of pork', Genet. Mol. Res., 12, 3383–3390, doi: 10.4238/2013.September.4.4, PMID: 24065679

[25] Giannenas I. Grigoriadou K. Sidiropoulou E. Bonos E. Cheilari A. Vontzalidou A.. (2021), Untargeted UHPLC-MS metabolic profiling as a valuable tool for the evaluation of eggs quality parameters after dietary supplementation with oregano, thyme, sideritis tea and chamomile on brown laying hens. Metabolomics, 17:51, doi: 10.1007/s11306-021-01801-7, PMID: 34021818

[26] Rocchetti G. Becchi P. P. Salis L. Lucini L. Cabiddu A. (2022), Impact of pasture-based diets on the untargeted metabolomics profile of Sarda sheep Milk. Foods, 12:143, doi: 10.3390/foods12010143, PMID: 36613358 PMC9818515

[27] Wang H. Xia P. Lu Z. Su Y. Zhu W. (2023), Time-restricted feeding affects transcriptomic profiling of hypothalamus in pigs through regulating aromatic amino acids metabolism. J. Sci. Food Agric., 103:1578–1587, doi: 10.1002/jsfa.12256, PMID: 36207281

[28] Zhou X. Liu Y. Zhang L. Kong X. Li F. (2021), Serine-to-glycine ratios in low-protein diets regulate intramuscular fat by affecting lipid metabolism and myofiber type transition in the skeletal muscle of growing-finishing pigs. Anim. Nutr. 7:384–392. doi: 10.1016/j.aninu.2020.08.011 34258426 PMC8245814

[29] Wang X. Wei H. Cao J. Li Z. He P. (2015), 'Metabolomics analysis of muscle from piglets fed low protein diets supplemented with branched chain amino acids using HPLC-high-resolution MS', Electrophoresis, 36, 2250–2258, doi: 10.1002/elps.201500007, PMID: 25820777

[30] Barboriak J. J. Krehl W. A. Cowgill G. R. (1957), 'Pantothenic acid requirement of the growing and adult rat', J. Nutr., 61, 13–21, doi: 10.1093/jn/61.1.13 13406604

[31] Qian Y. Li X. F. Zhang D. D. Cai D. S. Tian H. Y. Liu W. B. (2015), 'Effects of dietary pantothenic acid on growth, intestinal function, anti-oxidative status and fatty acids synthesis of juvenile blunt snout bream Megalobrama amblycephala', PLoS One, 10:e0119518, doi: 10.1371/journal.pone.0119518, PMID: 25781913 PMC4362765

[32] Wu G. Y. (2010), 'Functional amino acids in growth, reproduction, and health', Adv. Nutr., 1, 31–37, doi: 10.3945/an.110.1008, PMID: 22043449 PMC3042786

[33] Perry R. J. Camporez J. P. G. Kursawe R. Titchenell P. M. Zhang D. Perry C. J.. (2015), 'Hepatic acetyl CoA links adipose tissue inflammation to hepatic insulin resistance and type 2 diabetes', Cells, 160, 745–758, doi: 10.1016/j.cell.2015.01.012, PMID: 25662011 PMC4498261

