# Supplementary material for: Low-protein diets supplemented with glycine improves pig growth performance and meat quality: An untargeted metabolomic analysis
Source: Front Vet Sci. 2023 Apr 18;10:1170573. doi: 10.3389/fvets.2023.1170573 (PMC10153625; doi:10.3389/fvets.2023.1170573)

**Table S1. Dietary composition and nutrient level.**

| **Dietary treatment** | | | |
| --- | --- | --- | --- |
| **Composition** | ND | LPD | LPDG |
| **Corn** | 66.88 | 80.84 | 81.31 |
| **Soybean meal** | 23.90 | 11.75 | 10.5 |
| **Wheat bran** | 6.00 | 3.00 | 3.00 |
| **Soybean oil** | 0.88 | 1.44 | 1.90 |
| **calcium hydrophosphate** | 0.50 | 0.60 | 0.60 |
| **Limestone** | 0.54 | 0.53 | 0.53 |
| **NaCl** | 0.30 | 0.30 | 0.30 |
| **Premix*** | 1.00 | 1.00 | 1.00 |
| **L-lysine** | 0 | 0.37 | 0.42 |
| **DL-methionine** | 0 | 0.03 | 0.05 |
| **L-threonine** | 0 | 0.11 | 0.13 |
| **L-tryptophan** | 0 | 0.03 | 0.03 |
| **L-serine** | - | - | - |
| **L-glycine** | - | - | 0.57 |
| **Total** | 100.00 | 100.00 | 100.00 |
| **Analyzed value（%）** | | | |
| **DE（MJ/kg）** | 14.21 | 14.23 | 14.20 |
| **Crude protein** | 16.04 | 12.03 | 12.05 |
| **SID Lys** | 0.73 | 0.73 | 0.74 |
| **SID Ser** | 0.72 | 0.48 | 0.48 |
| **SID Gly** | 0.61 | 0.40 | 0.96 |
| **SID Thr** | 0.52 | 0.47 | 0.46 |
| **SID (Met＋Cys)** | 0.51 | 0.42 | 0.43 |
| **Total Ca** | 0.51 | 0.50 | 0.50 |
| **Total P** | 0.45 | 0.40 | 0.39 |
| **Available P** | 0.20 | 0.20 | 0.19 |

**Table S2.** Productive data of the growing-finishing pigs1.

| Index | ND | LPD | LPDG |
| --- | --- | --- | --- |
| IBW, kg | 59.2±1.2 | 59.6±1.4 | 59.7±0.9 |
| FBW, kg | 96.0±2.6 | 93.2±2.9 | 95.7±3.0 |
| ADG, g | 855±17^a^ | 777±15^b^ | 839±31^a^ |
| ADFI, kg | 2.22±0.09 | 2.25±0.08 | 2.42±0.06 |
| F:G | 2.59±0.08^b^ | 2.89±0.08^a^ | 2.90±0.14^a^ |

IBW=initial body weight; FBW=ﬁnal body weight; ADG= average daily weight gain; ADFI= average daily feed intake; F:G=the ratio of feed to gain.

a, b Mean values within a row with unlike superscript letters were signiﬁcantly different (P < 0.05). n=8.

^1^ ND, pigs fed a normal crude protein (16%) diet; LPD, pigs fed a low crude protein (12%) diet generated by decreasing the content of soybean meal; LPDG, pigs fed a low crude protein (12%) diet with serine-to-glycine ratio 1:2, generated by decreasing the content of soybean meal and adding 0.57% glycine.

**Table S3.** Significantly altered metabolites in LT muscle determined in the postmortem conversion of muscle to meat. Red meat increased and green means decreased in metabolite abundance.

| **Metabolites** | LPD vs ND | | | | | | | | LPDG vs LPD | | | |
| --- | --- | --- | --- | --- | --- | --- | --- | --- | --- | --- | --- | --- |
|  | 0.75h | | 4h | | 24h | | 0.75h | | 4h | | 24h | |
|  | FC | p value | FC | p value | FC | p value | FC | p value | FC | p value | FC | p value |
| ***Carbohydrate metabolites*** |  |  |  |  |  |  |  |  |  |  |  |  |
| D-Glucose 6-phosphate | 1.757 | 0.007 |  |  |  |  |  |  |  |  |  |  |
| Glyceraldehyde 3-phosphate | 0.112 | 0.028 |  |  |  |  |  |  |  |  |  |  |
| 3-phosphoglyceric acid | 0.019 | 0.001 |  |  |  |  | 10.46 | 0.009 | 0.016 | 0.000 |  |  |
| ***Amino acids and peptides*** |  |  |  |  |  |  |  |  |  |  |  |  |
| N-Stearoyl-L-tyrosine | 7.056 | 0.000 |  |  |  |  |  |  |  |  |  |  |
| Capryloylglycine | 1.890 | 0.006 |  |  |  |  |  |  |  |  |  |  |
| L-Aspartic acid | 3.075 | 0.000 | 2.533 | 0.003 |  |  |  |  |  |  | 0.488 | 0.004 |
| Leucyltryptophan | 3.259 | 0.000 | 3.762 | 0.000 |  |  |  |  |  |  |  |  |
| Leu-Leu | 4.245 | 0.000 | 7.512 | 0.000 |  |  |  |  |  |  |  |  |
| Isoleucylasparagine | 2.733 | 0.000 | 2.914 | 0.003 |  |  |  |  |  |  |  |  |
| Methionylleucine | 4.275 | 0.000 | 2.869 | 0.001 |  |  |  |  |  |  |  |  |
| Phe-Asn | 2.301 | 0.000 | 2.359 | 0.011 |  |  |  |  |  |  |  |  |
| Thr-Asp | 2.210 | 0.000 | 2.589 | 0.001 |  |  |  |  |  |  |  |  |
| Val-Ser | 1.663 | 0.008 |  |  |  |  |  |  |  |  | 0.378 | 0.019 |
| Leu-Gln | 2.761 | 0.003 |  |  |  |  |  |  |  |  |  |  |
| Ala-Tyr | 1.805 | 0.011 |  |  |  |  |  |  |  |  | 0.338 | 0.040 |
| Leu-arg | 3.176 | 0.002 |  |  |  |  |  |  |  |  |  |  |
| Leu-Val | 2.786 | 0.003 |  |  |  |  |  |  |  |  | 0.408 | 0.050 |
| Alanylleucine | 1.781 | 0.045 |  |  |  |  |  |  |  |  | 0.220 | 0.049 |
| Lys-Leu | 1.915 | 0.036 |  |  |  |  |  |  |  |  |  |  |
| N-Decanoylglycine | 2.692 | 0.005 |  |  |  |  |  |  |  |  |  |  |
| N-Acetylornithine |  |  | 2.136 | 0.027 |  |  | 0.611 | 0.047 |  |  | 0.597 | 0.036 |
| D-(+)-Proline |  |  | 1.870 | 0.001 |  |  |  |  |  |  |  |  |
| L-(-)-Asparagine |  |  | 4.503 | 0.000 |  |  |  |  |  |  |  |  |
| Taurine |  |  | 2.461 | 0.000 |  |  |  |  |  |  |  |  |
| Glycine |  |  | 4.736 | 0.000 |  |  |  |  |  |  |  |  |
| Threonine |  |  | 2.480 | 0.000 |  |  |  |  |  |  |  |  |
| DL-Glutamine |  |  | 3.834 | 0.000 |  |  |  |  |  |  |  |  |
| L-(-)-Serine |  |  | 3.047 | 0.000 |  |  |  |  |  |  |  |  |
| L-(+)-Citrulline |  |  | 2.366 | 0.000 |  |  |  |  |  |  |  |  |
| Tricine |  |  | 2.954 | 0.000 |  |  |  |  |  |  |  |  |
| L-Pyroglutamic acid |  |  | 4.075 | 0.000 |  |  |  |  |  |  |  |  |
| S-Adenosyl-L-homocysteine |  |  | 2.920 | 0.000 |  |  |  |  |  |  |  |  |
| Hypotaurine |  |  | 3.679 | 0.000 |  |  |  |  |  |  |  |  |
| Gly-Leu |  |  | 4.409 | 0.001 |  |  |  |  |  |  |  |  |
| N-Acetyl-L-carnosine |  |  | 2.654 | 0.000 |  |  |  |  |  |  |  |  |
| Aspartyl-L-proline |  |  | 2.245 | 0.000 |  |  |  |  |  |  |  |  |
| L-Alanyl-L-glutamine |  |  | 2.512 | 0.000 |  |  |  |  |  |  |  |  |
| N6,N6,N6-Trimethyl-L-lysine |  |  |  |  | 1.539 | 0.005 |  |  |  |  | 0.600 | 0.004 |
| N-lauroylglycin |  |  |  |  | 1.583 | 0.032 |  |  |  |  |  |  |
| Myristoyl Glycine |  |  |  |  | 1.696 | 0.017 |  |  |  |  | 0.602 | 0.024 |
| N6,N6,N6-Trimethyl-L-lysine |  |  |  |  | 1.539 | 0.005 |  |  |  |  | 0.600 | 0.004 |
| N-lauroylglycine |  |  |  |  | 1.583 | 0.032 |  |  |  |  |  |  |
| Myristoyl Glycine |  |  |  |  | 1.696 | 0.017 |  |  |  |  | 0.602 | 0.024 |
| N6,N6,N6-Trimethyl-L-lysine |  |  |  |  | 1.539 | 0.005 |  |  |  |  | 0.600 | 0.004 |
| N-lauroylglycine |  |  |  |  | 1.583 | 0.032 |  |  |  |  |  |  |
| Myristoyl Glycine |  |  |  |  | 1.696 | 0.017 |  |  |  |  | 0.602 | 0.024 |
| N-Acetyl-1-aspartylglutamic acid |  |  |  |  |  |  |  |  |  |  | 0.385 | 0.000 |
| Gly-l-pro |  |  |  |  |  |  |  |  |  |  | 0.552 | 0.043 |
| g-Glutamylcysteine |  |  |  |  |  |  |  |  |  |  | 0.551 | 0.000 |
| L-gamma-Glutamyl-L-aspartic acid |  |  |  |  |  |  |  |  |  |  | 0.631 | 0.012 |
| Leu-Gly-Pro |  |  |  |  |  |  |  |  |  |  | 0.281 | 0.016 |
| beta-Aspartylaspartic acid |  |  |  |  |  |  |  |  |  |  | 0.639 | 0.002 |
| L-(-)-Threonine |  |  |  |  |  |  |  |  |  |  | 0.599 | 0.047 |
| L-(-)-Methionine |  |  |  |  |  |  |  |  |  |  | 0.616 | 0.022 |
| DL-Arginine |  |  |  |  |  |  |  |  |  |  | 0.635 | 0.010 |
| ***Purine and Cofactors*** |  |  |  |  |  |  |  |  |  |  |  |  |
| Adenosine 5'-monophosphate （AMP） | 9.865 | 0.000 |  |  |  |  | 0.366 | 0.012 |  |  |  |  |
| Adenosine diphosphate (ADP) | 2.935 | 0.026 |  |  |  |  |  |  |  |  |  |  |
| Uridine monophosphate (UMP) | 3.503 | 0.008 |  |  |  |  |  |  |  |  |  |  |
| Guanosine triphosphate (GTP) | 0.074 | 0.033 |  |  |  |  |  |  |  |  |  |  |
| β-Nicotinamide mononucleotide (NMN) |  |  | 3.887 | 0.000 |  |  |  |  |  |  |  |  |
| Nicotinamide adenine dinucleotide (NAD+) |  |  | 2.851 | 0.001 |  |  |  |  |  |  |  |  |
| Hypoxanthin |  |  | 2.005 | 0.004 |  |  |  |  |  |  |  |  |
| Deoxyuridine monophosphate (dUMP) |  |  | 2.503 | 0.000 |  |  |  |  |  |  |  |  |
| 5,6-Dihydrothymidine |  |  | 2.759 | 0.000 |  |  |  |  |  |  |  |  |
| Cytosine |  |  | 3.492 | 0.000 |  |  |  |  |  |  |  |  |
| Adenine |  |  | 5.365 | 0.029 |  |  |  |  |  |  |  |  |
| ***Lipid*** |  |  |  |  |  |  |  |  |  |  |  |  |
| Acetyl-CoA | 0.355 | 0.002 |  |  |  |  |  |  |  |  | 0.468 | 0.003 |
| Erucic acid | 2.581 | 0.012 |  |  |  |  |  |  | 0.198 | 0.000 | 0.086 | 0.007 |
| 15S-hydroxyeicosatrienoic acid | 2.274 | 0.000 |  |  |  |  |  |  |  |  | 1.588 | 0.017 |
| 12-Hydroxyoctadecanoic acid | 2.001 | 0.004 |  |  |  |  |  |  |  |  |  |  |
| ω-9-Hydroperoxyarachidonic acid | 1.843 | 0.000 | 2.407 | 0.002 |  |  |  |  |  |  | 2.564 | 0.000 |
| Linoleyl carnitine | 5.729 | 0.002 |  |  |  |  |  |  |  |  |  |  |
| O-oleoylcarnitine | 3.607 | 0.010 |  |  |  |  |  |  |  |  |  |  |
| (2E)-hexadecenoylcarnitine | 2.845 | 0.025 |  |  |  |  |  |  |  |  |  |  |
| 3, 5-Tetradecadiencarnitine | 2.954 | 0.011 | 3.450 | 0.042 |  |  |  |  |  |  |  |  |
| 1-heptadecanoyl-sn-glycero-3-phosphocholine |  |  |  |  |  |  | 1.914 | 0.008 |  |  |  |  |
| L-alpha-Glycerylphosphorylcholine |  |  |  |  |  |  | 2.320 | 0.001 |  |  | 1.866 | 0.003 |
| 1-oleoyl-sn-glycero-3-phosphoethanolamine |  |  |  |  |  |  | 2.613 | 0.003 |  |  | 1.734 | 0.002 |
| 1-Linoleoyl-sn-glycero-3-phosphocholine |  |  |  |  |  |  | 2.407 | 0.005 |  |  |  |  |
| 1-[(9Z)-hexadecenoyl]-sn-glycero-3-phosphocholine |  |  |  |  |  |  | 1.674 | 0.019 |  |  |  |  |
| 1-(1Z-hexadecenyl)-sn-glycero-3-phosphocholine |  |  |  |  |  |  | 2.359 | 0.007 |  |  | 2.157 | 0.002 |

**Table S4. The pathway analysis of the identified metabolites affected in the LPD group compared to the ND group. Based on the selected metabolites, the global metabolic disorders of the most relevant pathways induced by LPD were revealed using the MetaboAnalyst 4.0.**

| Metabolism pathway name | Raw p | -log(10)p | FDR | Impact |
| --- | --- | --- | --- | --- |
| Phenylalanine, tyrosine and tryptophan biosynthesis | 0.028113 | 1.5511 | 0.91534 | 0.5 |
| Synthesis and degradation of ketone bodies | 0.035028 | 1.4556 | 0.91534 | 0 |
| Linoleic acid metabolism | 0.035028 | 1.4556 | 0.91534 | 1 |
| Ubiquinone and other terpenoid-quinone biosynthesis | 0.062244 | 1.2059 | 0.91534 | 0 |
| Phenylalanine metabolism | 0.068938 | 1.1615 | 0.91534 | 0 |
| Arginine biosynthesis | 0.095281 | 1.021 | 0.91534 | 0 |
| Butanoate metabolism | 0.10176 | 0.99242 | 0.91534 | 0 |
| Terpenoid backbone biosynthesis | 0.12095 | 0.91741 | 0.91534 | 0 |
| Citrate cycle (TCA cycle) | 0.13353 | 0.87443 | 0.91534 | 0.03668 |
| beta-Alanine metabolism | 0.13976 | 0.85463 | 0.91534 | 0.05597 |
| Pyruvate metabolism | 0.14595 | 0.83581 | 0.91534 | 0.15397 |
| Propanoate metabolism | 0.15209 | 0.81789 | 0.91534 | 0.01269 |
| Lysine degradation | 0.16427 | 0.78444 | 0.91534 | 0 |
| Glycolysis / Gluconeogenesis | 0.1703 | 0.76879 | 0.91534 | 0.04169 |
| Glutathione metabolism | 0.18224 | 0.73936 | 0.91534 | 0 |
| Inositol phosphate metabolism | 0.19402 | 0.71216 | 0.91534 | 0 |
| Glyoxylate and dicarboxylate metabolism | 0.20565 | 0.68688 | 0.91534 | 0.00794 |
| Biosynthesis of unsaturated fatty acids | 0.22844 | 0.64122 | 0.91534 | 0 |
| Pyrimidine metabolism | 0.24515 | 0.61057 | 0.91534 | 0.03727 |
| Fatty acid degradation | 0.24515 | 0.61057 | 0.91534 | 0.18092 |
| Fatty acid elongation | 0.24515 | 0.61057 | 0.91534 | 0.25661 |
| Valine, leucine and isoleucine degradation | 0.25064 | 0.60094 | 0.91534 | 0.02836 |
| Tryptophan metabolism | 0.2561 | 0.59158 | 0.91534 | 0 |
| Tyrosine metabolism | 0.26153 | 0.58248 | 0.91534 | 0.13972 |
| Fatty acid biosynthesis | 0.2881 | 0.54045 | 0.94763 | 0.00213 |
| Aminoacyl-tRNA biosynthesis | 0.29332 | 0.53267 | 0.94763 | 0 |
| Steroid hormone biosynthesis | 0.46338 | 0.33406 | 1 | 0.01032 |

**Table S5. The pathway analysis of the identified metabolites affected in the LPDG group compared to the LPD group. Based on the selected metabolites, the global metabolic disorders of the most relevant pathways induced by LPDG were revealed using the MetaboAnalyst 4.0.**

| Metabolism pathway name | Raw p | -log(10)p | FDR | Impact |
| --- | --- | --- | --- | --- |
| D-Glutamine and D-glutamate metabolism | 0.00172 | 2.764472 | 0.134 | 0.5 |
| Glutathione metabolism | 0.00319 | 2.496209 | 0.134 | 0 |
| Biosynthesis of unsaturated fatty acids | 0.00658 | 2.181774 | 0.184 | 1 |
| Arginine biosynthesis | 0.00988 | 2.005243 | 0.207 | 0 |
| Alanine, aspartate and glutamate metabolism | 0.0374 | 1.427128 | 0.616 | 0 |
| Phenylalanine, tyrosine and tryptophan biosynthesis | 0.044 | 1.356547 | 0.616 | 0 |
| Arginine and proline metabolism | 0.0652 | 1.185752 | 0.685 | 0 |
| Nitrogen metabolism | 0.0653 | 1.185087 | 0.685 | 0 |
| Ubiquinone and other terpenoid-quinone biosynthesis | 0.0964 | 1.015923 | 0.814 | 0.03668 |
| Aminoacyl-tRNA biosynthesis | 0.0981 | 1.008331 | 0.814 | 0.05597 |
| Phenylalanine metabolism | 0.107 | 0.970616 | 0.814 | 0.15397 |
| Butanoate metabolism | 0.156 | 0.806875 | 0.991 | 0.01269 |
| Nicotinate and nicotinamide metabolism | 0.156 | 0.806875 | 0.991 | 0 |
| Histidine metabolism | 0.165 | 0.782516 | 0.991 | 0.04169 |
| Pantothenate and CoA biosynthesis | 0.193 | 0.714443 | 1 | 0 |
| beta-Alanine metabolism | 0.211 | 0.675718 | 1 | 0 |
| Porphyrin and chlorophyll metabolism | 0.288 | 0.540608 | 1 | 0.00794 |
| Glyoxylate and dicarboxylate metabolism | 0.305 | 0.5157 | 1 | 0 |
| Arachidonic acid metabolism | 0.336 | 0.473661 | 1 | 0.03727 |
| Tyrosine metabolism | 0.38 | 0.420216 | 1 | 0.18092 |

**Figure S1.** Principle component analysis (PCA) for total lipid content and sensory attributes of LT muscle from pigs receiving normal, low protein, and low protein supplemented with glycine diets.


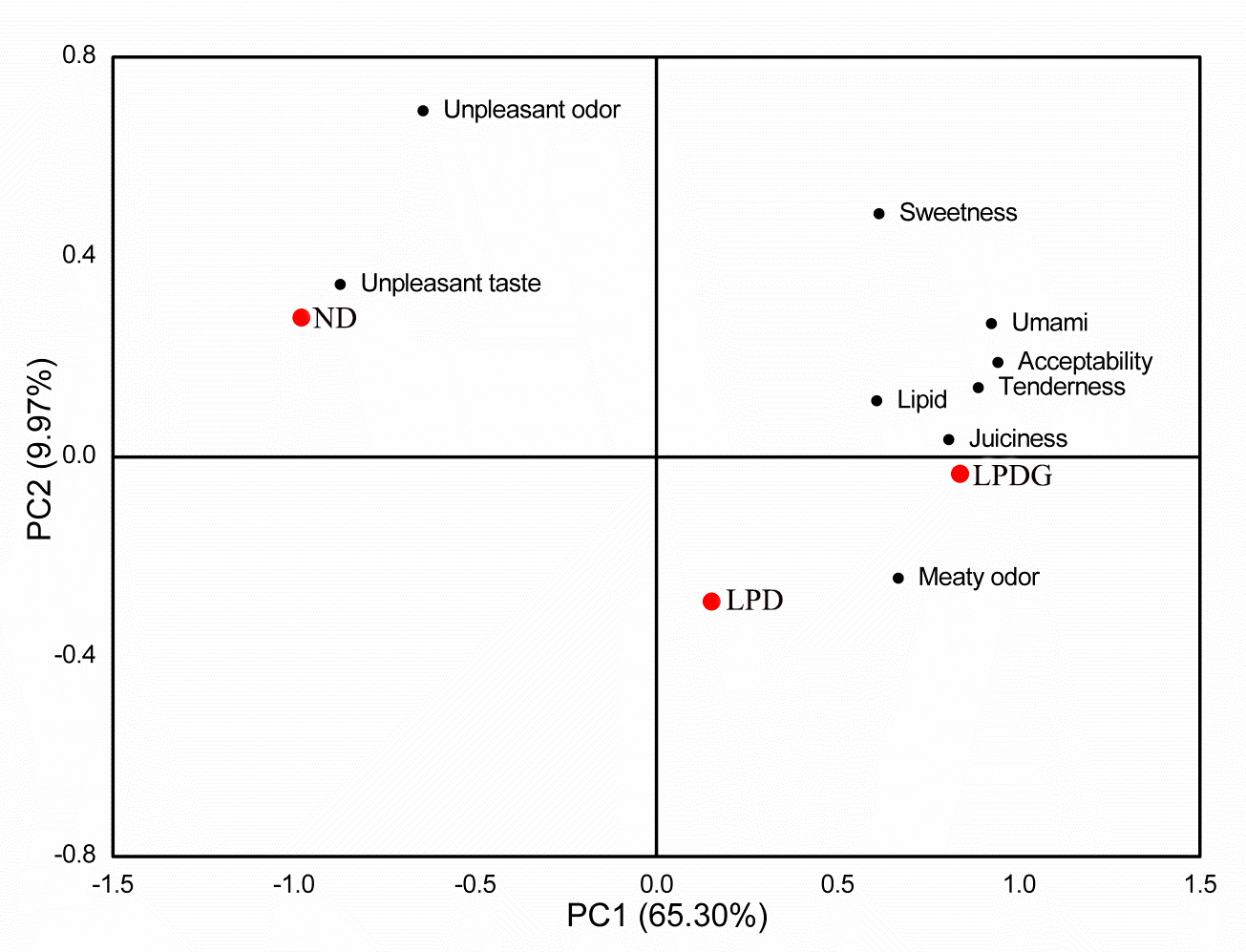


**Figure S2.** Hierarchical cluster analysis (HCA) of metabolites from LT muscle of pigs receiving a normal, low protein, or low protein supplemented with glycine diet. The Heatmap shows changes in metabolite abundance, where red and blue colors represent increased and decreased metabolites relative to the median metabolic level. Muscle samples are displayed as columns and labeled with dietary treatment and pig number.





**Figure S3.** Volcano plots of multiple matrices for screening differential metabolites between low protein diet and the normal diet, low protein diet supplemented with glycine and low protein diet at 0, 0.75, 4 and 24 h PM, respectively. Each circle represents one metabolite. The point size of big and small indicate the VIP values of the top 30% and the remaining 70%. Differential metabolites marked in the plot were determined by combining restrictions of three dimensions: p < 0.05, VIP > 1.0 and FC > 1.5 or FC < 0.667. Metabolites in red were up-regulated and metabolites in green were down-regulated, p < 0.05.


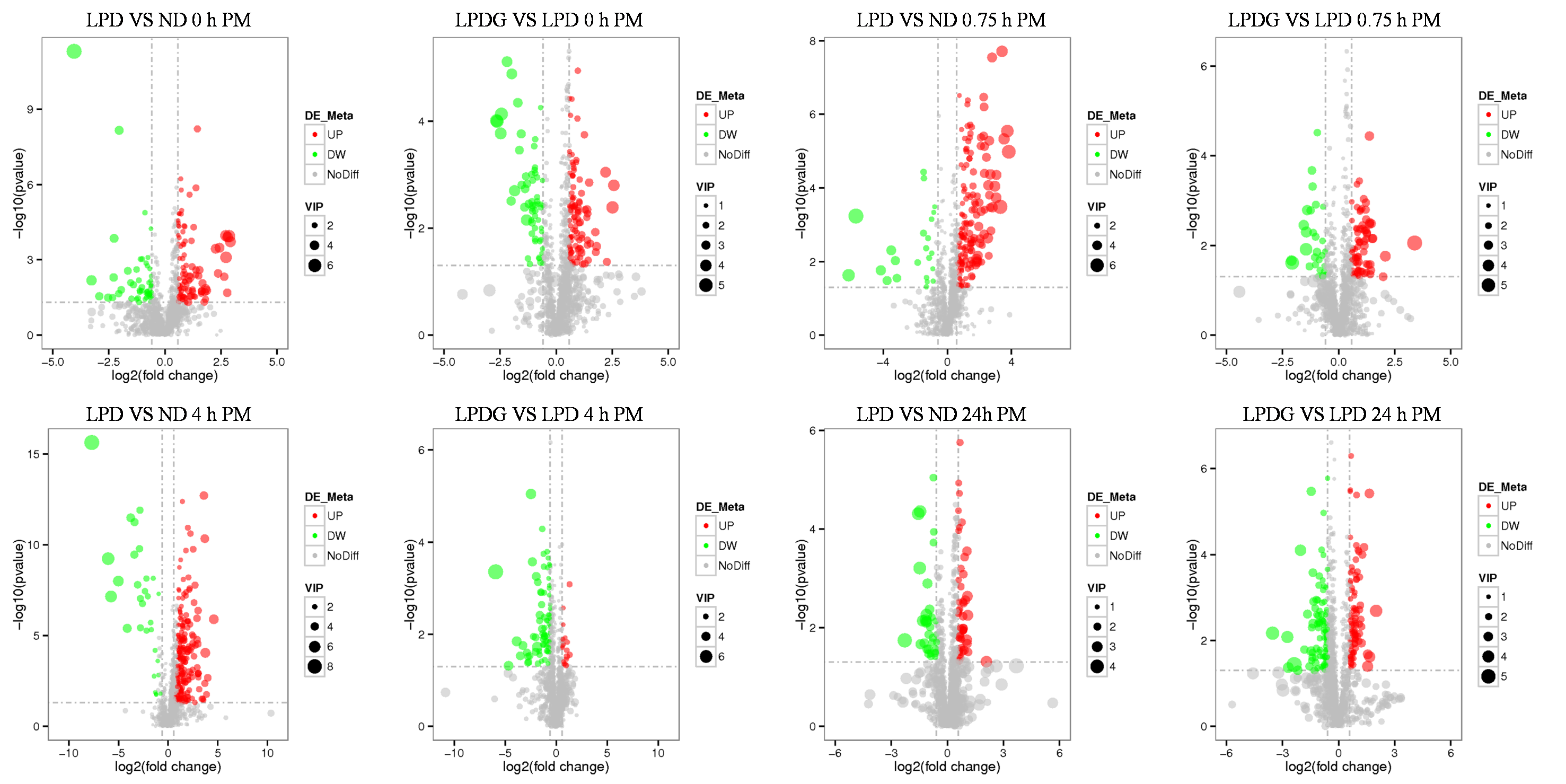

Supplement: Supplementary file 1 [file Data_Sheet_1.doc]
